# Mediating Role of Rumination and Negative Affect in the Effect of Mind-Wandering on Symptoms in Patients With Obsessive-Compulsive Disorder

**DOI:** 10.3389/fpsyt.2021.755159

**Published:** 2021-10-14

**Authors:** Pengchong Wang, Wenwen Cao, Tao Chen, Jian Gao, Yifan Liu, Xiangyun Yang, Fanqiang Meng, Jing Sun, Zhanjiang Li

**Affiliations:** ^1^Beijing Key Laboratory of Mental Disorders, National Clinical Research Centre for Mental Disorders, Beijing Anding Hospital, Capital Medical University, Beijing, China; ^2^Advanced Innovation Center for Human Brain Protection, Capital Medical University, Beijing, China; ^3^Brain and Mind Center, The University of Sydney, Sydney, NSW, Australia; ^4^School of Psychology, The University of Sydney, Sydney, NSW, Australia; ^5^Menzies Health Institute Queensland and School of Medicine and Dentistry, Griffith University, Brisbane, QLD, Australia

**Keywords:** obsessive-compulsive disorder, mind wandering, rumination, negative affect, mediation analysis

## Abstract

To explore the relationship between negative affect, mind-wandering, rumination and obsessive-compulsive symptoms, 100 patients with obsessive-compulsive disorder and 100 healthy controls were assessed using the Obsessive-Compulsive Inventory, the Beck Anxiety Inventory, the Beck Depression Inventory, the Mind Wandering Scale and the Ruminative Response Scale. The results show that (i) patients diagnosed with obsessive-compulsive disorder displayed higher obsessive-compulsive symptoms, negative affect, mind-wandering and rumination compared with healthy controls; (ii) negative affect, mind-wandering and rumination were positively correlated with the severity of obsessive-compulsive symptoms; (iii) mind-wandering predicted the severity of obsessive-compulsive symptoms (both directly and indirectly); (iv) rumination and negative affect mediated the relationship between mind-wandering and obsessive-compulsive symptoms. The results preliminarily reveal the relationship between mind-wandering and psychopathological obsessive-compulsive symptoms, providing a reference for exploring novel psychological treatments for obsessive-compulsive disorder.

## Introduction

Obsessive-compulsive disorder (OCD) is characterised by frequent and severe unwanted intrusive thoughts and repetitive or ritualised behaviours (1). While some obsessions are related to external factors, OCD involves frequent spontaneous and unwanted thoughts, also known as unintentional mind-wandering (MW), that abruptly into one's consciousness without any identifiable evoking stimuli (2, 3). Cognition models of OCD (4, 5) often focus on how thoughts can transform into psychopathological obsessions and mental distress. Individuals who misinterpret or ruminate on normal mental phenomena are more likely to develop obsessive-compulsive symptoms (OCS) (6). While MW has been associated with OCS, the relationship between them is not well understood.

Dysfunctional MW may be common to multiple mental health disorders (7). The inward focus associated with MW may increase the likelihood of self-absorbed thinking, which can elicit negative thoughts (8). For example, people who frequently engage in unintentional MW are more likely to report symptoms of depression, anxiety and stress (9). Thus, dysfunctional MW may enhance negative thoughts and negative affect, increasing the risk of psychopathological symptoms.

## Background

### MW and OCD Symptomatology

According to a dynamic framework of thought (7), MW is a special case of spontaneous thinking that is less constrained and more goal oriented compared with rumination and obsessive thinking. MW is predictive of stress (10), depression (11) and anxiety (12) and has become a prominent topic in cognitive psychology (13). MW also provides a new perspective, namely that spontaneous thoughts may play a role in the cause and maintenance of OCS (14). MW is closely related to OCS in non-clinical samples (6, 14–16). For example, Seli, Risko, Purdon and Smilek (14) found that MW is correlated with different dimensions of OCS. In addition, MW is considered a specific type of out-of-context thought. Fradkin and Huppert (2) found that OCS is associated with a higher probability of recalling negative (v. non-negative) out-of-context thoughts, implying that MW may be a risk factor for OCS. However, to date, few studies have explored how the wandering mind affects OCS in clinical samples.

### Mediating Effect of Rumination and Negative Affect in the Relationship Between MW and OCS

Although some studies have found that MW is correlated with OCS, not all spontaneous thoughts are unique to OCD (17). Understanding when spontaneous thoughts are not adaptive may clarify their role in the aetiology of OCD, shedding light on the still unexplained association between psychopathology and risks to mental health.

According to the MW–perseverative cognition continuum hypothesis, when MW loses its adaptivity and becomes rigid and inflexible, it may result in a repetitive spiral of homogeneous negative thoughts (e.g., excessive rumination and low self-esteem) and lead to cognitive vulnerability (18, 19). The spontaneous thought model posits that abnormal spontaneous thoughts can reduce cognitive control and turn into rumination (18). MW may also augment repetitive thinking (20). Marchetti, Koster (21) have demonstrated that internally oriented thoughts during rest may encourage a ruminative self-focus, in turn worsening negative mood.

According to the cognition model of OCD, maladaptive rumination may play a role in the onset and maintenance of psychopathological OCS. Recent evidence suggests that people with OCD can experience severe maladaptive rumination, which has been positively correlated with different dimensions of OCS in non-clinical samples (6, 15, 22). Maladaptive rumination is a key symptom in people with OCD, who feel responsible for its consequences, thus excessively focus on their thoughts (23). Several studies suggest that ruminating on one's intrusive thoughts may result in abnormal appraisals of naturally occurring spontaneous thoughts, resulting in the persistence of negative affect (6, 24, 25).

Rumination is a complex cognition vulnerability factor that may mediate the relationship between naturally occurring thoughts and OCS. In light of the MW psychopathology hypothesis, we assume that MW in itself a maladaptive process but may become a risk factor for OCS if it manifests as rigid and inflexible rumination, leading to negative appraisals of naturally occurring thoughts, obsessions and distress.

People with OCD generally experience negative affect comorbidities such as anxiety, depression or disgust (26, 27). This negative affect is directly related to not only individuals' wellbeing and life satisfaction (28) but also OCS such as repetitive intrusive thoughts (29). Negative affect is a potential obstacle to OCD treatment and may impede a patient's ability to change, augment patient distress and predict worse treatment outcomes (30).

MW is a critical factor in negative affect states. For example, involuntary MW is highly correlated with symptoms of anxiety and depression (9, 31). Several studies have shown that MW may be indirectly associated with negative affect via rumination, which may lead to anxiety and depression through a variety of mechanisms, often referred to as transdiagnostic factors (32, 33). Marchetti, Van de Putte and Koster (11) found that daydreaming can result in depressive symptoms via excessive self-focus and rumination. Smallwood, O'Connor and Heim (34) found that off-task thoughts are more frequently correlated with depression in high ruminators compared with low ruminators. In addition, Marchetti, Koster and De Raedt (21) reported that higher internal focus during resting states predicts increased levels of rumination, resulting in more negative moods. It has been suggested that MW may induce abnormal rumination (11), meaning that people with OCD are more inclined to catastrophise with negative misinterpretations and intrusive thoughts, thus increasing the negative affect experience and further worsening OCS (24, 25).

Previous studies on the relationships between OCS, rumination and MW have mostly been limited to non-clinical samples (2, 14, 16, 35). This study aims to explore (i) MW, rumination and negative affect in people with clinical OCD compared with healthy controls (HCs) and (ii) whether rumination and negative affect have a chain mediating effect on the relationship between MW and OCS. We hypothesise that (i) people with OCD will experience a higher frequency of MW and more severe rumination and negative affect compared with HCs and (ii) higher MW will predict more severe rumination and negative affect, in turn predicting more severe OCS. Shedding light on the underlying processes may increase the understanding of conditions that increase the likelihood of MW related to OCS.

## Materials and Methods

### Participants

In total, 100 patients with OCD (55 females and 45 males) were recruited from the outpatient clinics of Beijing Anding Hospital at Capital Medical University. The patients met the following inclusion criteria: (i) OCD diagnosed according to the Mini-International Neuropsychiatric Interview (MINI) (36); (ii) aged between 18 and 45 years; and (iii) no history of neurological or other major physical disease, Axis I psychiatric disorders (other than OCD), Axis II personality disorders or drug or alcohol abuse. The Yale-Brown Obsessive-compulsive Scale (Y-BOCS) (37) mean score was 20.01 ± 7.496 (cut-off point = 16.0) (obsession subscale = 10.67 ± 4.454; compulsion subscale = 9.30 ± 4.949). Comorbid depression and anxiety symptoms were measured using the Beck Depression Inventory-II (BDI-II) (14.465 ± 10.806) and Beck Anxiety Inventory (BAI) (13.538 ± 10.584) respectively, with low overall average results. Of the 100 participants, 37 (37%) had never been prescribed medications or had stopped taking medications for at least 4 weeks before participating in this study.

For the control group, 100 healthy participants (54 females and 46 males) w**ere** recruited via advertising and met the same criteria as the OCD group; however, participants in the control group were excluded if they had been diagnosed with an Axis I psychiatric disorder according to the Diagnostic and Statistical Manual of Mental Disorders (fourth edition) (DSM-IV).

Both groups were matched for age (28.58 ± 6.365 years for patients with OCD, 27.98 ± 5.53 years for HCs, *t* = 0.712 *p* = 0.478), gender (χ^2^ = 0.020, *p* = 0.887) and education level (χ^2^ = 1.308, *p* = 0.727).

### Procedure

All participants were voluntary and signed the informed consent form. The study was approved by the Research Ethics Committee of Beijing Anding Hospital, Capital Medical University, Beijing, China (Ethic no. 2019, scientific research no. 02). Participants were individually interviewed by a trained researcher and completed self-reported measures in a quiet area of the neuropsychological laboratory at the Beijing Key Laboratory of Mental Disorders. Each session lasted approximately 45 min.

### Measures

#### Diagnosis and OCS

The researcher used MINI to diagnose OCD and Y-BOCS to evaluate the severity of OCD in participants who met the DSM-IV OCD diagnostic criteria. The Obsessive-Compulsive Inventory–Revised (OCI-R) (38) was used to measure self-reported OCS in the OCD and HC groups (cut-off point = 21). The OCI-R evaluates OCS experienced in the past month on a 5-point scale (0 = *not at all*; 4 = *extremely*). The Chinese version of OCI-R shows good internal consistency (Cronbach's α = 0.84) and test–retest reliability (*r* = 0.96) in both clinical and non-clinical samples (39).

#### Negative Affect

The BAI was used to measure anxiety in participants. The inventory evaluates the degree to which 21 different symptoms affected participants during the past week on a 4-point Likert scale (0 = *not at all*; 1 = *mild, little trouble*; 2 = *moderate, uncomfortable but tolerable*; and 3 = *severe, can scarcely be endured*). The total score is calculated by adding the scores of all and ranges from 0 to 63 points (cut-off score for clinical anxiety is 16) (40). The Chinese version of the BAI has demonstrated excellent internal consistency (Cronbach's α = 0.95) (41).

The Beck Depression Inventory-II (BDI-II) was used to assess depressive symptoms. This is a popular 21-item self-reported measure of the presence and severity of depression symptoms during the past week. The BDI-II is based on a 4-point Likert scale (0 = *absent* to 3 = *severe*). The total score is calculated by adding the scores of all and ranges from 0 to 63 points (cut-off score of clinical depression is 18.5) (42). The Chinese version of the BDI-II has demonstrated good internal consistency (Cronbach's α = 0.94) and test–retest coefficients (*r* = 0.55) (43).

#### Rumination

The Ruminative Response Scale (RRS) is an effective and popular method of measuring rumination. Yang, Ling and Xiao (44) produced a Chinese version of the RRS (RRS-C), which shows good internal consistency (Cronbach's α = 0.88) and mean internal item correlation coefficients (ranging from 0.29 to 0.32) in depressed adult patients (45). The RRS-C contains 21 items based on a 4-point scale (1 = *never*, 2 = *sometimes*, 3 = *often*, 4 = *always*), which measures the frequency of the given symptoms. The higher the score (from 21 to 84 points), the higher the level of rumination.

#### MW

The Mind Wandering Scale (MWS), taken from the Imaginal Processes Inventory (46), contains 12 items that reflect the frequency of MW characteristics in daily life based on a 5-point scale (1 = *completely inconsistent*, 2 = *comparatively inconsistent*, 3 = *difficult to determine*, 4 = *comparatively consistent*, 5 = *completely consistent*). The total score ranges from 12 to 60 points, with a higher score indicating more frequent MW. Carciofo, Du, Song and Zhang (47) produced a Chinese version of the MWS (MWS-C), which demonstrates good internal reliability (Cronbach's α = 0.851; test–retest reliability = 0.718).

### Statistical Analysis

IBM SPSS Statistics 26.0 software was used for statistical analysis. All general clinical data were described as mean ± standard deviation. The independent samples *t*-test and chi-squared test were used to compare the continuous and category variables, respectively, in the OCD and HC groups. To prevent Type I and Type II errors caused by unsatisfied presuppositions in the classic parameter test, the bias-corrected percentile bootstrap method was used to analyse the mediation effect. The mediation model was tested using PROCESS 3.3 (48) with a sample size of 5,000. A bias-corrected non-parametric percentile with a 95% confidence interval was selected as the sampling method, all *p*-values were two-tailed, and statistical significance was set at 0.05.

## Results

### Group Difference

The results of the independent sample *t*-test showed that OCS, negative affect, rumination and MW scores in the OCD group were all significantly higher than those in the HC group (see Table 1).

**Table 1:** Differences between obsessive-compulsive symptoms, negative affect, mind-wandering and rumination between groups.

	**OCD (*n* = 100)**	**HC (*n* = 100)**	***t***	***p***
BAI	13.538 ± 10.584	2.160 ± 3.320	10.258	< 0.001
BDI-II	14.465 ± 10.806	3.100 ± 3.948	9.879	< 0.001
OCI-R	21.540 ± 14.799	2.300 ± 3.572	12.638	< 0.001
MWS-C	38.530 ± 10.105	29.600 ± 8.430	6.786	< 0.001
RRS-C	44.170 ± 12.635	30.625 ± 7.150	9.330	< 0.001

*OCD, obsessive-compulsive disorder; HC, healthy control; BAI, Beck Anxiety Inventory; BDI-II, Beck Depression Inventory-II; OCI-R, Obsessive-Compulsive Inventory–Revised (Chinese version); MWS-C, Mind Wandering Scale–Chinese; RRS-C, Ruminative Response Scale–Chinese*.

### Correlation Between Negative Affect, MW, Rumination and OCS

After controlling for gender, age and education level, partial correlation analysis revealed that negative affect, MW, rumination and OCS were significantly and positively correlated (see Table 2).

**Table 2:** Correlation between negative affect, mind-wandering, rumination and obsessive-compulsive symptoms (*N* = 200).

	**OCI-R**	**MWS-C**	**RRS-C**	**BAI**
MWS-C	0.544^**^	—	—	—
RRS-C	0.725^**^	0.615^**^	—	—
BAI	0.652^**^	0.501^**^	0.638^**^	—
BDI-II	0.729^**^	0.579^**^	0.743^**^	0.757^**^

*BAI, Beck Anxiety Inventory; BDI-II, Beck Depression Inventory-II; OCI-R, Obsessive-Compulsive Inventory–Revised (Chinese version); MWS-C, Mind Wandering Scale–Chinese RRS-C, Ruminative Response Scale–Chinese;*

** *p < 0.01*.

Chain mediation effects of rumination and negative affect on the relationship between MW and OCS

### Collinearity and Normality Test

A collinearity test was used to prevent multicollinearity among independent variables. OCS was the dependent variable, while age, gender, education, anxiety, depression, MW and rumination were the independent variables. The results show that all tolerance values were >0.10 (gender = 0.978, age = 0.942, education = 0.966, depression = 0.301, anxiety = 0.401, MW = 0.578, rumination = 0.383), and all variance inflation factor values were <5 (gender = 1.022, age = 1.062, education = 1.036, depression = 3.319, anxiety = 2.494, MW = 1.731, rumination = 2.608). In the normality test, the skewness values of all variables ranged from 0.097 to 1.610, while kurtosis values ranged from −0.512 to 2.363.

### Chain Mediation Analysis

To investigate whether spontaneous thoughts mediated the linear relationship between negative affect and OCS, we followed the PROCESS 3.3 procedure for testing mediator effects. Given that there were two mediators, Model 6 was selected, and gender, age and education levels were included as covariate variables.

First, we established the mediator model for anxiety and OCS. Hierarchical regression analysis (see Table 3) showed that age significantly predicted anxiety (β = 0.201, *p* < 0.05), MW significantly predicted rumination (β = 0.727, *p* < 0.001) and anxiety (β = 0.176, *p* < 0.01), and rumination significantly predicted anxiety (β = 0.423, *p* < 0.001). Age (β = 0.286, *p* < 0.05), MW (β = 0.175, *p* < 0.05), rumination (β = 0.570, *p* < 0.001) and anxiety (β = 0.408, *p* < 0.001) all had a significant predictive effect on OCS.

**Table 3:** Hierarchical regression analysis between MW and OCS: Rumination and Anxiety as mediators.

**Variables**	**Rumination**		**Anxiety**		**OCS**	
	**β**	***t***	**β**	***t***	**β**	***t***
Gender	−1.399	−1.006	−0.186	−0.177	−1.412	−1.074
Age	−0.105	−0.893	0.201	2.267^*^	0.286	2.543^*^
Education	−0.457	−0.670	−0.348	−0.676	−0.451	−0.698
MW	0.727	10.766^***^	0.176	2.744^**^	0.175	2.141^*^
Rumination			0.423	7.833^***^	0.570	7.358^***^
Anxiety					0.408	4.548^***^
*R*	0.621		0.664		0.780	
*R^2^*	0.386		0.441		0.609	
*F*	30.664^***^		30.631^***^		50.053^***^	

*MW, mind-wandering; OCS, obsessive-compulsive symptoms*.

* *p < 0.05,*

** *p < 0.01,*

*** *p < 0.001*.

**Figure 1:**
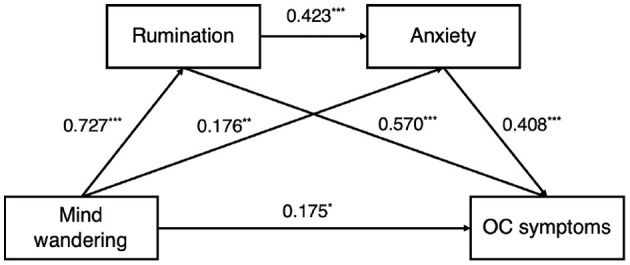
Mediation effect path of rumination and anxiety between mind-wandering and obsessive-compulsive (OC) symptoms. ***p < 0.001.

Results of the mediation effect analysis (see Figure 1) showed that MW directly predicted OCS (direct effect = 0.175, *p* = 0.033), and anxiety and rumination partially mediated this relationship (indirect effect = 0.612). Specifically, the mediation effect consisted of three indirect effects:

- Path 1: MW → rumination → OCS (indirect effect = 0.414)
- Path 2: MW → anxiety → OCS (indirect effect = 0.072)
- Path 3: MW → rumination → anxiety → OCS (indirect effect = 0.126).

None of the bootstrap 95% confidence intervals contained 0, indicating that all three paths had a significant indirect effect.

Next, we established a mediator model for MW and OCS. Hierarchical regression analysis (see Table 4) showed that MW had a significant positive predictive effect on rumination (β = 0.727, *p* < 0.001); both MW (β = 0.194, *p* < 0.001) and rumination (β = 0.501, *p* < 0.001) significantly predicted depression; and age (β = 0.338, *p* < 0.01), rumination (β = 0.453, *p* < 0.001) and depression (β = 0.577, *p* < 0.001) significantly predicted OCS.

**Table 4:** Hierarchical regression analysis between MW and OCS: Rumination and Depression as mediators.

**Variables**	**Rumination**		**Depression**		**OCS**	
	**β**	***t***	**β**	***t***	**β**	***t***
Gender	−1.399	−1.006	0.382	0.409	−1.708	−1.341
Age	−0.105	−0.893	0.051	0.652	0.338	3.145^**^
Education	−0.457	−0.670	−0.587	−1.284	−0.254	−0.405
MW	0.727	10.766^***^	0.194	3.395^***^	0.136	1.692
Rumination			0.501	10.457^***^	0.453	5.544^***^
Depression					0.577	5.890^***^
*R*	0.621		0.762		0.796	
*R^2^*	0.386		0.580		0.633	
*F*	30.664^***^		53.580^***^		55.446^***^	

MW, mind-wandering; OCS, obsessive-compulsive symptoms. *p < 0.05,

** *p < 0.01,*

*** *p < 0.001*.

**Figure 2:**
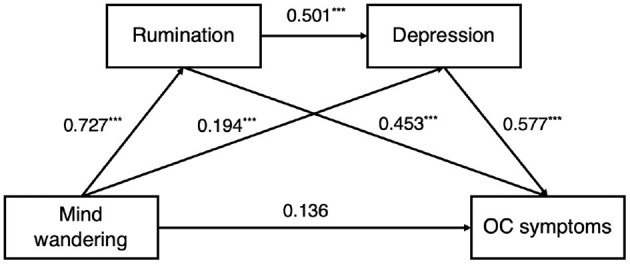
Mediation effect path of rumination and depression between mind-wandering and obsessive-compulsive (OC) symptoms. ***p < 0.001.

Results from the mediation effect analysis (see Figure 2) showed that MW did not directly predict OCS (direct effect = 0.136, *p* = 0.092), but rumination and depression fully mediated this relationship (indirect effect = 0.652). Specifically, the mediation effect consisted of three indirect effects:

- Path 1: MW → rumination → OCS (indirect effect = 0.330)
- Path 2: MW → depression → OCS (indirect effect = 0.112)
- Path 3: MW → rumination → depression → OCS (indirect effect = 0.210).

None of the bootstrap 95% confidence intervals contained 0, indicating that all three paths had a significant indirect effect.

## Discussion

In the present study, OCD patients showed more severe negative affect and abnormal thought activity compared with HCs, which is consistent with previous research findings (49). Further, correlation analysis showed that MW and rumination were significantly and positively correlated with OCS, which is again consistent with previous studies (6, 14), suggesting that OCS is closely related to spontaneous thoughts.

MW was conceptualised as the failure of executive control, including a reduced ability to bring one's consciousness back to a goal-directed state and enable internally generated task-related thoughts to flourish (50). OCD is also characterised by impairments in executive functions (e.g., response inhibition) and sustained attention (51). A recent biological model of differences in executive functioning demonstrated that durable representations in the prefrontal cortex (e.g., a strong image) are associated with weaker prefrontal–posterior connectivity, resulting in impaired inhibition and flexibility in shifting attention (52). This may explain why MW results in decreased integrative functioning and attention deficits (35), implying that MW may impair executive control, in turn inducing and maintaining OCS.

Further, MW and OCS may share a common neurological mechanism. As a mental baseline, MW is considered an outcome of the default mode network (DMN) (53), while OCD has been strongly associated with altered connectivity in the DMN (54). The DMN plays an important role in monitoring the external environment; thus, an abnormal activation of the DMN in OCD may lead to attention lapses and a more internal or external focus. Given this, the findings of this study—the mediating effect of MW on the relationship between clinical OCS and cognitive thought and neurobiological processes—may guide future research.

The mediation analyses conducted in this study highlighted specific factors that may explain the severity of OCS, namely MW, rumination and negative affect. MW had a significant direct effect on OCS; that is, the higher the frequency of MW, the higher the severity of OCS. Further, rumination and negative affect had a chain mediating effect on the relationship between MW and OCS, revealing that the psychological influence of MW on OCS depends on the level of rumination and negative affect.

Notably, MW indirectly influenced OCS through the following three mediation pathways: (i) MW → rumination → OCS, (ii) MW → negative affect → OCS and (iii) MW → rumination → negative affect → OCS. Individuals who experience MW may focus on internal thoughts, leading to rumination. Negative and catastrophic ruminations on naturally occurring thoughts may lead to compulsive behaviours or neutralisation to suppress these unexpected thoughts and prevent the expected disastrous consequences, exacerbating the severity of OCS.

Given that almost one-third of patients experiencing their first episode of OCD also exhibit symptoms of anxiety and depression (55), negative affect may reinforce the occurrence and maintenance of OCS in several ways, including exacerbating functional disability (56), predicting poor quality of life (49, 57) and weakening attention control (58). In our assumption model, MW affects OCS not only via rumination but also via negative affect, either directly or indirectly. In particular, rumination and depression play a mediating role in the relationship between MW and OCS severity. Although the relationship between MW and negative affect is still unclear, MW may also result in anxiety or depression in some conditions. MW has been characterised as “coming out of nowhere”, resulting in people with OCD feeling threatened or anxious (2). Rumination also results in maladaptive cognition, leading patients to interpret intrusive thoughts in a more negative and catastrophic way and amplifying their distress (59), and inhibits emotional processing (60). This is supported by findings that rumination mediates the relationship between MW and depression (11).

### Limitations

The current study has several limitations. First, while it preliminarily revealed that MW affects OCS via rumination and negative affect, it was unable to specify whether abnormal MW and rumination lead to more severe OC-related beliefs and cognitive distortions in OCD patients. In addition, although we used self-reporting to examine subjects' recent negative affect and spontaneous thoughts, this does not demonstrate cause and effect between spontaneous thoughts and negative affect. Therefore, we suggest that the characteristics of MW during negative emotional states need to be examined. An emotion-evoking and experience-sampling paradigm may be used to explore the characteristics of negative affect and spontaneous thoughts and their relationship with OCS. Finally, although PROCESS 3.3 uses the bias-corrected percentile bootstrap method to test for mediation effects and has lower requirements for sample size and data morphology, rendering it suitable for smaller samples, our results should be further validated in a large sample of patients, and longitudinal studies are needed to explore further the causal relationship between MW and OCS.

### Conclusion

This study showed that MW not only has a direct impact on OCS but also increases rumination and negative affect, leading individuals into more negative, catastrophic and uncontrolled processing of their naturally occurring thoughts, in turn inducing more distressing OCS. These findings contribute to the OCD literature and have both research and clinical implications. Regulating MW and rumination in OCD patients through, for example, mindfulness-based cognitive behaviour therapy may help reduce OCS, abnormal MW activity (61) and negative affect (62).

## Data Availability Statement

The raw data supporting the conclusions of this article will be made available by the authors, without undue reservation.

## Ethics Statement

The studies involving human participants were reviewed and approved by Research Ethics Committee of Beijing Anding Hospital, Capital Medical University, Beijing, China. The patients/participants provided their written informed consent to participate in this study.

## Author Contributions

PW: designed the study, performed literature searches, drafted the manuscript, critically reviewed, and revised the manuscript. WC: performed literature searches, collected dated, and drafted the manuscript. TC: conceptualised the study, reviewed, and revised the manuscript. JG: collected data and revised the manuscript. YL: recruit, assessment, and data collected. XY and FM: both contributed to collected data. ZL and JS: both contribute to conceptualised and designed the study, reviewed, and revised the manuscript, and as co-corresponding authors. All authors contributed to the article and approved the submitted version.

## Funding

National Natural Science Foundation of China (No. 81271493). Beijing Municipal Administration of Hospitals Incubating Program (code: PX2020075). Beijing Municipal Administration of Hospitals Clinical Medicine Development of Special Funding Support (code: XMLX202129).

## Conflict of Interest

The authors declare that the research was conducted in the absence of any commercial or financial relationships that could be construed as a potential conflict of interest.

## Publisher's Note

All claims expressed in this article are solely those of the authors and do not necessarily represent those of their affiliated organizations, or those of the publisher, the editors and the reviewers. Any product that may be evaluated in this article, or claim that may be made by its manufacturer, is not guaranteed or endorsed by the publisher.
